# Exploring lncRNA-Mediated Mechanisms in Muscle Regulation and Their Implications for Duchenne Muscular Dystrophy

**DOI:** 10.3390/ijms26136032

**Published:** 2025-06-24

**Authors:** Abdolvahab Ebrahimpour Gorji, Zahra Roudbari, Kasra Ahmadian, Vahid Razban, Masoud Shirali, Karim Hasanpur, Tomasz Sadkowski

**Affiliations:** 1 Department of Physiological Sciences, Institute of Veterinary Medicine, Warsaw University of Life Sciences, 02-787 Warsaw, Poland; abdolvahab_ebrahimpourgorji@sggw.edu.pl; 2 Department of Animal Science, Faculty of Agriculture, University of Jiroft, Jiroft 78671-55311, Iran; roudbari.zahra@ujiroft.ac.ir; 3 Department of Animal Science, Faculty of Agriculture, Ferdowsi University of Mashhad, Mashhad 91779-48974, Iran; k.ahmadian@areeo.ac.ir; 4 Agri-Food and Biosciences Institute, Belfast BT9 5PX, UK; vahid.razban@afbini.gov.uk (V.R.); masoud.shirali@afbini.gov.uk (M.S.); 5 School of Biological Sciences, Queen’s University Belfast, Belfast BT9 5DL, UK; 6 Department of Animal Science, Faculty of Agriculture, University of Tabriz, Tabriz 51666-16471, Iran; karimhasanpur@tabrizu.ac.ir

**Keywords:** skeletal muscle, DMD, LncRNA, gene regulation

## Abstract

Duchenne muscular dystrophy (DMD) manifests as a hereditary condition that diminishes muscular strength through the progressive degeneration of structural muscle tissue, which is brought about by deficiencies in the dystrophin protein required for the integrity of muscle cells. DMD is among four different types of dystrophinopathy disorders. Current studies have established that long non-coding RNAs (lncRNAs) play a significant role in determining the trajectory and overall prognosis of chronic musculoskeletal conditions. LncRNAs are different in terms of their lengths, production mechanisms, and operational modes, but they do not produce proteins, as their primary activity is the regulation of gene expression. This research synthesizes current literature on the role of lncRNAs in the regulation of myogenesis with a specific focus on certain lncRNAs leading to DMD increments or suppressing muscle biological functions. LncRNAs modulate skeletal myogenesis gene expression, yet pathological lncRNA function is linked to various muscular diseases. Some lncRNAs directly control genes or indirectly control miRNAs with positive or negative effects on muscle cells or the development of DMD. The research findings have significantly advanced our knowledge about the regulatory function of lncRNAs on muscle growth and regeneration processes and DMD diseases.

## 1. Introduction

Duchenne muscular dystrophy (DMD) is an X-linked recessive muscular dystrophy caused by pathogenic variants in the dystrophin gene (*DMD*), affecting patients from early childhood [1]. The majority of DMD patients aged 2–3 years old have muscle breakdown that leads to progressive weakness, more prominent initially in the proximal lower limbs, that rapidly leads to wheelchair dependence by early adolescence, as described in the natural history of the disease [2,3]. Deaths among DMD patients occur between 20 and 40 years because their muscles weaken to extreme debilitation. Thus, the primary cause of death is end-stage cardiomyopathy, often complicated by superimposed respiratory infections or acute episodes [4]. Various studies and sources have different reports concerning DMD prevalence as well as its incidence at birth. Current research suggests DMD affects 0.9 to 16.8 of 100,000 males, but birth prevalence studies suggest DMD occurs 1.5 to 28.2 times in 100,000 live-born male children. The numerous scientific estimates reflect the difficulty in estimating DMD incidence in males [5].

The pathogenetic variants of the *DMD* gene cause progressive deterioration of the skeletal muscle, the interplay of damage resulting from disrupted muscle fibers, and severe cycles of degeneration and regeneration, leading to progressive muscle atrophy and replacement with fibrotic and adipose tissues during the disease process [6]. The myogenesis of skeletal muscle is a multi-step process with various discrete stages characterized by the co-existence of myogenic regulatory factors (MRFs), which include *Myf5*, myogenic differentiation antigen (*MyoD)*, myogenin (*MyoG)*, and *Mrf-4*, as well as myosin heavy chain (*Mhc*) [7]. From a pathogenetic perspective, dystrophin deficiency leads to progressive muscle deterioration, inflammation, and pro-oxidative/mitochondrial stress [8,9]

LncRNA has now been acknowledged as a crucial factor directly interacting with multiple myogenic regulatory factors. The long non-coding RNA class comprises non-coding RNA species that exceed 200 nucleotides in length [4,10,11]. These RNA species play imperative roles by modulating gene expression at transcriptional and post-transcriptional levels [12,13]. LncRNAs differ from mRNAs as they do not have an open reading frame (ORF), having around 2.8 exons, whereas proprotein-coding RNA has 11 exons and low abundance [14]. LncRNAs can indirectly regulate gene expression in three ways: direct activation of transcription factors, miRNA sponge, enzymatic recruitment to genomic loci, and sequence changes [15,16]. Research has shown that some non-coding RNAs control muscle growth and development, albeit their genetic analysis boundaries are poorly understood [17,18].

Due to their underuse in genetic analysis, these RNAs have not received much focus in genetic research on muscle development. The Pathways Studio Software Web Mammalian (V. 12.5.0.2, 2022) was used to filter out matching lncRNAs that potentially influence the development of DMD and their miRNA binding and genetic motifs, and available literature on DMD and lncRNA, for the current review. LncRNA is encoded by more than 56,946 genes (127,802 transcripts) but has little or no potential to produce protein [19]. In addition, lncRNAs are transcribed into some peptides with regulatory functions [20].

Based on high-throughput technologies and bioinformatics analyses, thousands of lncRNAs have been discovered in skeletal muscles, but only a few have been identified as functionally regulated (Table 1). Hou et al. examined the skeletal muscle transcriptome, detecting 322 lncRNAs. The identified lncRNAs regulate the gene function of *Csrp3*, *Myf6*, *Igfbp*, *Dcn*, *Map2k1*, *Spp1*, and *Acsl1*, which control skeletal muscle development and fatty acid metabolism [19]. The groups of lncRNAs bind proteins to affect the development and deterioration of muscles. *Metabolism-related lncRNAs:* Gm15441 controls insulin signal transmission while sustaining stable blood glucose levels in skeletal muscles. Pparα activation level rises during fasting periods, leading to Gm15441 gene expression elevation through Pparα-binding sites in its promoter [21]. The protein Gm15441 forms a complex with Txnip and decreases protein amounts, which results in reduced hepatic glucose production [22]. A Spearman correlation analysis showed 3110045C21Rik and Ddr2 expression occur together in type 2 diabetes mellitus (T2DM) patients, which indicates 3110045C21Rik regulates insulin-signaling mechanisms [23]. *Fibrosis-related lncRNAs:* Furthermore, the overexpression of 3110045C21Rik promotes the up-regulation of E-cadherin (Cdh1) while suppressing the expression of alpha-smooth muscle actin (Acta2) and Transforming Growth Factor Beta 1 (Tgfb1), key markers associated with the development and progression of fibrosis [24]. *Myogenesis and Apoptosis-related lncRNAs:* Recent literature identifies several lncRNAs related to skeletal muscle atrophy, including SYISL, Mir22hg, Myoparr, Pvt1, RP11-253E3.3, PRKG1-AS1, lncDLEU2, Chronos, lnc-ORA, lncMUMA, Atrolnc-1, H19, HOTAIR, Malat1, PVT1, Gm15441, 3110045C21Rik, Gm20743, LncEDCH1, ZFP36L2-AS, LncIRS1, SMUL, lnc-mg, and MYH1G-AS. These lncRNAs regulate key processes in muscle atrophy through diverse mechanisms, including modulation of protein synthesis, ubiquitin-proteasome-mediated protein degradation, and myogenic differentiation [25]. A Weighted Gene Co-expression Network Analysis (WGCNA) of goat skeletal muscle in developmental stages revealed that LNC_011371 (targets 74 genes, including Mb and Clic5, that are up-regulated after birth), LNC_007561 (targets Tcf4 that regulates myogenesis), and LNC_001728 (binds to S100A4 gene and promotes cardiomyocyte production and increases myocardial cell number by inhibiting apoptosis) involve in muscle structure formation, p53-signaling pathways, and MAPK-signaling pathways [26].

## 2. LncRNAs in Muscle Development

The myogenic differentiation process benefits from Ppp1r1b-lncRNA positive regulatory action. The Ppp1r1b and polycomb repressive complex 2 (PRC2) interaction stimulates the transcription of myogenic transcription factors in a mouse C2C12 myoblast cell line and human skeletal myoblasts. The absence of Ppp1r1b leads *Ezh2* to enhance its binding activities on the regulatory regions of these myogenic transcription factors. The suppression of *MyoD*, Myogenin, and *Tbx5*, along with sarcomere proteins, becomes very evident. The research indicates that Ppp1r1b functions as a regulatory factor during the early stages of muscle development and heart formation [27]. One of the essential lncRNAs that regulates some transcription factors and miRNAs is the myocardial infarction-associated transcript (Miat). This lncRNA can indirectly regulate some biological processes in muscle, such as cell proliferation, cell differentiation, regeneration, and myogenesis. For instance, Miat upregulates the expression of the transcription factor *Foxo1* by sponging miR-139-5p [38], and this gene regulates myogenic differentiation and growth, muscle atrophy, and glycemic properties [39,40]. Moreover, Miat indirectly regulates the expression of *Zeb1* by acting as a sponge and repressing miR-150. Expression of *Zeb1* has been reported in numerous tissues, including the immune system, and significantly regulates muscle and lymphoid differentiation [41]. Notably, both *Zeb1* and *Zeb2* are pivotal elements that modulate the TGF-β-mediated signaling pathway through their interaction with *Smad* proteins to facilitate the recruitment of co-activators or co-repressors [42]. Current studies demonstrate that Miat operates directly to control three transcription factors named *Dnmt1*, *Dnmt3a*, and *Dnmt3b*. Research findings indicate that decreasing Miat levels limits attachment to these transcription factors [28]. Phytohemagglutinin stimulation increased apoptosis and inhibited cell proliferation in response to suppression of the *Dnmt1* protein. Evidence shows that the gene modifier exerts substantial control over essential biological operations within immune cells [43]. A feedback loop between *Dnmt3b* and miR-125b controls vascular smooth muscle cell responses to homocysteine exposure [44]. Studies demonstrate that *MyoG* attaches to the promoter of this lncRNA to cause its expression activation. It was also shown that enhanced lncRNA-1700113A16RIK expression accelerates the differentiation process of MuSC cells, while decreased expression adversely affects the function of cells. Moreover, this lncRNA directly interacts with the 3′UTR of myogenic genes, including the myogenic transcription factor *Mef2d*, subsequently enhancing its translation [29].

Investigations into lncRNA expression patterns during skeletal muscle development identified ten lncRNAs, which displayed maximal correlations with known protein-coding genes. Hub lncRNAs consisting of lnc-22988, lnc-372289, and lnc-482286 exhibited positive correlation while connecting with 156 protein-coding genes, including the crucial skeletal muscle developmental genes *Acta1*, *Eno3*, *Myl1*, *Myom1*, *Myoz1*, *Neb*, *Ryr1*, and *Tnnc2*. The three lncRNAs are linked to various genetic elements that potentially control muscle proliferation and differentiation and global developmental pathways [30]. The expression of miR-675-3p and miR-675-5p increases due to H19 regulation in skeletal muscle cells, and these miRNAs reduce *Smad1*, *Smad5*, and *Cdc6* gene activity to drive both skeletal muscle differentiation and regeneration [31]. Moreover, an increase in H19 results in the higher expression of dual-specificity phosphatase 27 (*Dusp27*) at the posttranscriptional level, promotes AMPK pathway activity in muscle cells, and stimulates glucose uptake and mitochondrial biogenesis. The decline of this lncRNA expression in human and mouse muscles leads to muscle insulin resistance, while high concentrations of H19 can enhance muscle insulin sensitivity [32]. In the embryonic stage, lncRNAs such as H19, Alien, and Miat are identified to have a key regulatory function. However, in cardiac development, their role is unclear, and H19 displays a dynamic epicardial, myocardial, and endocardial expression during cardiac development [45].

Several lines of evidence demonstrate that Chronos plays a pivotal role in hypertrophy. Using inducible *Akt1* transgenic mice to investigate age-related lncRNAs revealed that Chronos inhibits *Akt1* and hypertrophic growth. Studies in vivo and in vitro show that Chronos inhibition’s effect on the *Bmp*-signaling pathway in general [46] and the *Bmp7* ligand specifically [47], leading to increased myofiber hypertrophy [48]. Furthermore, the *Bmp* gene is essential for developing blood vessels required for steady muscle states [49].

## 3. LncRNA and miRNA—Working Together in Muscles

Skeletal muscle formation depends on lncRNAs and miRNAs, which operate within a complex regulatory system to control myogenesis. They regulate chromatin, activate transcription, act as miRNAs sponges, modulate RNA stability and translation, and, in some cases, encode micropeptides. MiRNAs regulate myogenic gene expression precisely, while lncRNAs, often functioning as sponges, modulate miRNA activity to fine-tune proliferation, differentiation, and regeneration. This coordinated regulation is critical for muscle development and holds promise for diagnosing and treating muscle diseases, such as muscular dystrophies [50]. A direct relationship exists between lncRNA and miRNA expression patterns, combining regulatory mechanisms across biological operations. The lncMgpf functions as an example by influencing the expression level of miR-135a-5p. Through this interaction, lncMgpf weakens the inhibitory effects of miR-135a-5p, consequently elevating the expression of *Mef2c*. This action further increases human antigen R (*HuR*)-mediated mRNA and regulates muscle development genes like *MyoD* and *MyoG* [51]. Another study shows that AK003290 in mice and its homologous lncRNA AK394747 in pigs and MT510647 in humans positively regulate *MyoD* expression and enhance myogenic differentiation of muscle cells [5]. During muscle differentiation in C2C12 cells, miR-487b regulates *Wnt5a*, a key player in myogenesis, and promotes muscle differentiation and regeneration. A study on lncRNA shows that muscle anabolic regulator 1 (*MAR1*) may regulate miR-487b and positively correlate with muscle differentiation. In addition, this lncRNA significantly enhanced mRNA and protein levels of myogenic markers (*MyoD*, *MyoG*, *Mef2c*, and *Myf5*), with highly expressed formation of myotubes in mouse skeletal muscle [33]. Moreover, overexpression of the intronic sense-overlapping lncRNA known as Syisl adversely affects the differentiation process of C2C12 cells. Elevated Syisl expression delays cellular differentiation while promoting cell proliferation [52]. Also, Syisl homologs in humans (designated hSyisl) and pigs (designated pSyisl) regulate myogenesis through interactions with *Ezh2* and regulate muscle atrophy and sarcopenia [34].

### 3.1. Cell Cycle and Differentiation Regulators

Specific lncRNAs function during the regulation of multiple myogenesis steps, and their depletion or dysregulation impairs cellular capacity to achieve cell cycle exit, resulting in aberrant proliferation. LncMyoD (Gm45923) is a regulatory lncRNA that binds directly to *Igf2*-mRNA-binding protein 2 (IMP2) during myoblast differentiation, downregulating the IMP2-mediated translation of proliferation-associated genes like *N-Ras* and *c-Myc* [53]. Meanwhile, miR-370-3p, which is directly targeted by lncMyoD, promotes myoblast proliferation and hinders myogenic differentiation of the C2C12 cell line [35]. Another lncRNA, Dum, follows the path of myoblast differentiation when stimulated by *MyoD*. Silencing *Dppa2* and suppressing the *MyoD–*Dum*–Dppa2* complex formation further augments myoblast differentiation and damage-induced muscle regeneration. A Dum knockdown could affect satellite cell activation, proliferation, or self-renewal capacity [36]. Furthermore, a network analysis of lncRNA-miRNA-gene interactions related to myogenesis led to the identification of lncIrs1, which regulates myoblast proliferation and differentiation [54]. LncRNA-Irs1 influences *Irs1* expression downstream of the Igf1-receptor-signaling pathway. Elevating lncIrs1 expression in breast muscle mitigates muscle atrophy and enhances muscle weight. Its overexpression boosts the phosphorylation of AKT within the IGF-1 pathway [52].

### 3.2. Atrophy Regulators

Research on lncRNA FKBP1C has shown that it is a developmental regulator of skeletal muscle tissue that prevents myoblast replication while supporting differentiation, mostly in fast and slow muscle fibers. Research on the *Myh1b* gene silencing primarily showed effects in fast muscle fibers. The lncRNA enhances *Myh1b* expression through cis-regulation, promoting protein stability, and influencing myoblast development [37]. The co-expression network analysis highlighted three important lncRNAs linked to skeletal muscle and atrophy, which are Ac004797.1, Prkg1-As1, and Grpc5d-As1. Research results demonstrated that the knockdown of Prkg1-As1 increased *MyoD*, *MyoG*, and *Mef2c* gene expression while reducing apoptosis in myoblast cells and the mortality rate [55].

The decoy activity of lncRNAs results in miRNA sequestration, which blocks their interaction with target mRNAs and disrupts miRNA-mRNA communication networks [47,56]. MiR-127 interferes with the retrotransposon-like one protein (Rtl1) sense transcript, reducing Rtl1 protein production. The protein is essential throughout muscle regeneration and present within regenerating and dystrophic muscle tissue [57]. Another research, by Liu et al. (2020), demonstrates that miR-324 limits C2C12 myoblast differentiation while accelerating intramuscular lipid accumulation through regulation by the lncRNAs Dum and Pm20d1 [58]. Moreover, it was shown that lncRNA-Miat functions as a direct target of miR-214, demonstrating that inhibition of this miRNA restores hepatocellular carcinoma cell proliferation and invasion by counteracting the suppression of Miat expression levels [59].

## 4. LncRNA in DMD Disease

**Figure 1:**
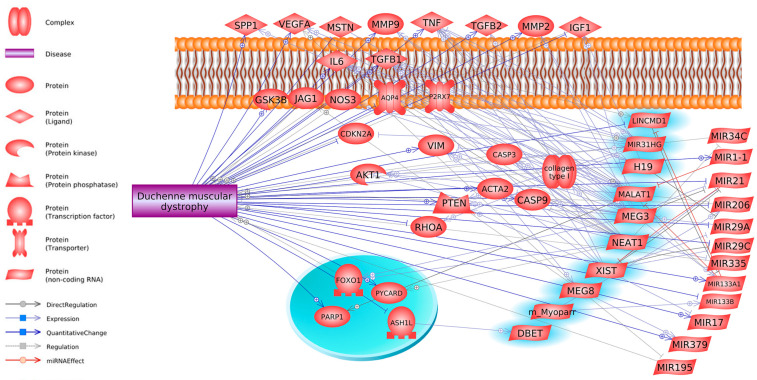
Interactions of lncRNAs and miRNAs in the context of DMD (Pathway Studio Mammalian Web). Blue markers indicate lncRNAs that, together with miRNAs, are involved in interactions with disease-related genes.

Atypical expression of lncRNAs is linked to diverse muscular disorders, most notably DMD, as outlined in Table 2. Current investigations have revealed that lncRNAs can regulate gene expression and translation, significantly influencing the progression of pathological states during cellular and muscular differentiation and development. Alterations in the expression of some lncRNAs are evident in the skeletal muscles of DMD patients (Figure 1).

Bovolenta et al. (2012) extensively investigated the *DMD* gene to elucidate how lncRNAs regulate gene expression and how nuclear lncRNAs modulate promoter regions to control muscle-specific isoform expression. Most regulatory lncRNA elements are found in intronic regions and nuclear spaces [60]. A detailed study based on bioinformatics identified Xist, Al132709, Linc00310, and Aldh1l1-As2 as key lncRNA regulators of DMD secondary processes, such as muscle degeneration and fibrosis. The diverse biological functions of these lncRNAs entail tumor-promoting capacity and ventricular septum development [61]. Dystrophin stabilizes the sarcolemma and is a scaffold for various intracellular signaling pathways, including nitric oxide synthase (NOS) signaling and the PI3K/Akt pathway [62]. LncRNAs, such as H19 and Malat1, are implicated in modulating these pathways. For instance, H19 can promote Akt1 expression, potentially counteracting muscle atrophy in dystrophic muscles, while lnc-31 influences myoblast differentiation by regulating myogenic gene expression [60,63]. These lncRNAs may act as upstream regulators or downstream effectors of dystrophin-dependent signaling, indicating a complex interplay that could amplify or mitigate the signaling defects caused by dystrophin deficiency [64].

**Table 2:** Control of select genes through lncRNA modulation.

LncRNA	Chr Location	Effect on Genes	Genes	Chr Location	References	DMD Effect on Gene	References
Meg3	14	positive	*Igf1*	12	[65]	positive	[66]
Meg3	14	negative	*Akt1*	14	[67]	positive	[68]
Meg3	14	negative	*Il6*	7	[69]	positive	[70]
Meg3	14	negative	*Mmp9*	20	[71]	positive	[72]
Meg3	14	negative	*Tgfb1*	19	[73]	positive	[74]
Meg3	14	negative	*Vegfa*	6	[75]	positive	[76]
Meg3	14	positive	*Casp3*	4	[77]	-	-
Meg3	14	positive	*Casp9*	1	[78]	positive	[79]
Meg3	14	positive	*Foxo1*	13	[80]	-	-
Meg3	14	positive	*Mmp2*	16	[81]	positive	[82]
Meg3	14	positive	*Pten*	10	[83]	positive	[84]
Neat1	11	negative	*Akt1*	14	[85]	positive	[68]
Neat1	11	negative	*Casp9*	1	[86]	positive	[79]
Neat1	11	positive	*Acta2*	10	[87]	positive	[88]
Neat1	11	positive	*Casp3*	4	[89]	-	-
Neat1	11	positive	*Foxo1*	13	[90]	-	-
Neat1	11	positive	*Il6*	7	[91]	positive	[70]
Neat1	11	positive	*Pycard*	16	[92]	-	-
Neat1	11	positive	*Tgfb1*	19	[93]	positive	[74]
Xist	X	positive	*Pten*	10	[94]	positive	[84]
Xist	X	positive	*Tgfb1*	19	[95]	positive	[74]
Xist	X	negative	*Tgfb2*	19	[96]	positive	[97]
Malat1	11	positive	*Aqp4*	18	[98]	negative	[99]
Malat1	11	negative	*Mmp2*	16	[100]	positive	[82]
Malat1	11	positive	*Akt1*	14	[101]	positive	[68]
Malat1	11	positive	*Mmp9*	20	[102]	positive	[72]
Malat1	11	positive	*Nos3*	7	[103]	positive	[104]
Malat1	11	positive	*Parp1*	1	[105]	positive	[106]
Lnc31	9	negative	*Gsk3b*	3	[107]	positive	[108]
Lnc31	9	positive	*Pten*	10	[109]	positive	[84]
H19	11	positive	*Igf1*	12	[110]	positive	[66]
H19	11	positive	*Akt1*	14	[111]	positive	[68]
H19	11	positive	*Il6*	7	[112]	positive	[70]
H19	11	positive	*Vegfa*	6	[113]	positive	[76]
Meg8	14	positive	*Jag1*	20	[114]	negative	[115]
Meg8	14	positive	*Vegfa*	6	[116]	positive	[76]
Dbet	4	positive	*Ash1l*	1	[117]	negative	[118]

For DMD research, a continued examination of lncRNAs occurred in the C2C12 cell line and human myoblasts. The study showed that lnc-31 exists at higher levels in DMD patient subjects than in individuals without DMD. The nuclear-based precursor molecule that produces miR-31 also creates the significant factor lnc-31, which guides the differentiation of precursor myoblasts. When lnc-31 is absent, the elevated expression of myogenin along with Atp2a1 causes rapid myogenic differentiation, which becomes difficult to reverse. This implies that lnc-31 is critical in controlling the transition from cell-cycle exit to terminal differentiation [119]. Another notable lncRNA in skeletal muscle regeneration and dystrophic muscles resides in the gene’s intron 44 (lncRNA44s2). This lncRNA exhibits expression during myogenesis in primary human myoblasts. It demonstrates activity linked to myogenesis, indicating its possible role in muscle differentiation and its potential as a disease-progression biomarker [63].

It is interesting to note that specific lncRNAs impact the progression of skeletal muscle atrophy. Physiological studies on different muscle atrophy models show depressed levels of lncMaat. Restoring or increasing lncMaat expression might then counteract multiple forms of atrophy. LncMaat inhibits miR-29b transcription through *Sox6* and simultaneously represses *Mbnl1* expression from the same regulatory module, leading to muscle atrophy regardless of miR-29b activity. *Mbnl1* overexpression shows excellent potential for preventing muscle atrophy [120]. The literature data show that Long Intergenic Non-Protein Coding RNA, Muscle Differentiation 1 (LincMD1), becomes vital in understanding the development of DMD, as its expression is significantly reduced in myoblasts from DMD patients compared to healthy controls. The reduced levels of LincMD1 are connected to the delayed expression and development of *Mhc* and myogenin muscle-specific markers in DMD tissue. The therapeutic potential of LincMD1 is demonstrated by its ability to enhance *Myog* and *Mef2c* expression in DMD patient-derived myoblasts, promoting myogenic differentiation [121].

Scientific evidence reveals a significant relationship between serine/threonine-protein kinase MRCK alpha (*Mrckα*) and α-synuclein (*Snca*) with lncRNA-H19. When *Mrckα* and *Snca* actively bind, they displace H19 from interacting with *DMD* gene segments to promote better myotube development and more efficient cell fusion in skeletal muscle cells. The administration of AGR-H19-gain-of-function enhances WT mouse muscle tissue development, metabolic performance, and muscle mass increase [120]. The lncRNA H19 accelerates muscle regeneration by activating the miR-675-3p and miR-675-5p, which regulate myogenic pathways [31]. The influence of specific non-muscle-specific lncRNAs shapes muscle proliferation and differentiation processes and contributes to both normal and pathological conditions. The cellular proliferation inhibitor named maternally expressed gene 3 (*Meg3*) functions to regulate muscle metabolism, along with glucose tolerance functions. Limited lncRNA Meg3 activity impairs glucose tolerance and negatively affects muscle metabolism [122,123]. According to Butchart et al. (2018), the expression of the *Meg3* gene remained the same between the atrophy disease model, mdx skeletal muscles, and normal C57 mouse muscle tissues [124].

**Figure 2:**
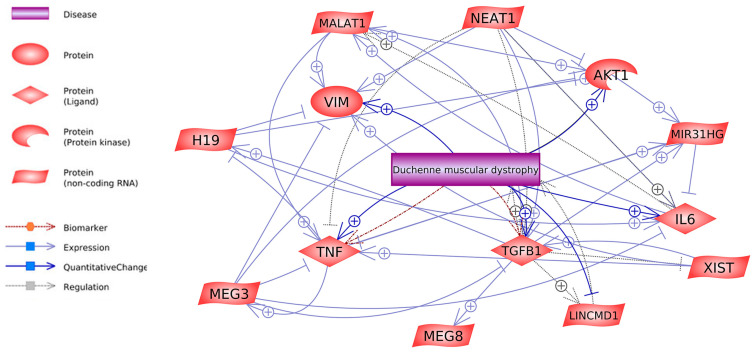
The interaction of lncRNAs with hub genes.

Many lncRNAs work together to control *Tnf* gene activity, which might become a diagnostic marker for DMD [125]. Studies demonstrate elevated *Tnf* gene expression levels in DMD patients compared to healthy samples [9,70]. This regulation involves a spectrum of lncRNAs, including Meg3, Xist, lnc-31 (Mir31hg), Neat1, and Malat1, culminating in intricate control over this key gene’s activity (see Figure 2).

A study showed that Meg3 overexpression decreased the pro-inflammatory cytokines *Tnf-α*, *Il-6*, and *Il-1β* levels [126]. The opposite results occur after silencing of Meg3 [127,128]. The increased expression level of Meg3 leads to diminished cell multiplication and reduced T-bet, Ifn-β, and Tnf-α levels. The reduction in these factors results in substantial increases in cell multiplication. The functions of lnc-31 and Neat1 in *Tnf-α* expression regulation match each other. The inhibitory action of lnc-31 on *Tnf-α* expression facilitates cell growth that might help reduce hypoxia-generated cell impairment. Neat1 exhibits anti-inflammatory properties that provide potential benefits against DMD inflammation when combined with associated treatments [129].

The expression profile of *Tnf-α* can be changed by several lncRNAs, such as Malat1 and Xist, because they activate the gene for Tnf-α production. Lowering of lncRNA Malat1 yields two primary effects on skeletal muscle cells. It leads to decreased *Il-6*, *Il-8*, *Tnf-α* serum levels, triggers cell death, and causes Akt-1 protein activation [130]. The reduction in Xist expression inhibits *Tnf-α* protein production and stops the Tnf-α/Rankl-signaling pathway activation process. The expression levels of *Tnf-α* significantly increase when Xist is overexpressed. Elevated levels of Xist following bone fractures hinder cell proliferation and differentiation, whereas reducing Xist expression promotes cell growth and regeneration [131]. The literature demonstrates that this lncRNA helps activate *Pten* by blocking the action of miR-17. Research has revealed that Xist silencing results in elevated levels of miR-17 in patients who experience type A aortic dissection [132]. The study of Yue et al. 2021 reported that *Pten* expression levels increased within the DMD and mdx mouse muscles, showing signs of muscular dystrophy. Reports indicate that Xist lncRNA indirectly supports the progression of DMD [84].

The clinical severity of DMD depends strongly on the functioning of the *Tgfb1* gene. Evaluation of *Tgfb1* expression enhances fibroblast multiplication and collagen synthesis while transforming fibroblasts into myofibroblasts [133]. The lncRNA molecules Neat1, Xist, and Meg3 maintain control over this gene. In molecular studies, in vitro experiments have shown that Xist can stimulate *Tgfb1* expression by upregulating miR-185 [134]. Neat1 stimulates *Tgfb1* overexpression through a competitive sponge action against miR-339-5p [135]. The RNA analysis demonstrates that Meg3 controls the cellular transition known as Epithelial-to-Mesenchymal Transition (EMT) through its ability to suppress *Tgfβ* signaling. Interestingly, inhibiting *Tgfβr1* or its downstream effectors, like *RhoA*, p38 MAPK, or *Snai2,* was proven to successfully reinstate facets of myogenic fusion and differentiation in vitro. The Meg3 functions as an inhibitor of *Tgfbi* or *Tgfb1* expression while integrating a modification of anti-myogenic EMT-promoting factors *Tgfβ*, *RhoA*, and *Snai2* in myoblasts and injured skeletal muscle [136] (Figure 2). The expression of Akt1 protein is upregulated through the regulatory actions of lncRNAs H19 and Malat1, which facilitate muscle hypertrophy in mdx mice [68].

## 5. How lncRNAs Can Control DMD Through miRNA and Gene Networks

Recent bioinformatics studies revealed distinct mechanisms by which Meg3 functions in its biological processes. The study shows that Meg3 functions as a miR-21-5p sponge, suggesting that this miRNA targets the 3′ untranslated region of the *Pten* sequence. Due to the overexpression of Meg3, *Pten* gene expression increases, leading to control of Pi3k/Akt signaling [137]. A third lncRNA, termed Neat1, reduces the expression levels of *Akt1*. The overexpressed miR-214 subsequently leads to elevated Pi3k, Akt, P-Akt, and Vegf level. The activating effect is achieved through *Pten* reduction. The expression of this specific lncRNA opposes the protective role of miR-214 on cerebral ischemia–reperfusion damage. By restoring *Pten* levels, the inhibition of Pi3k together with Akt, P-Akt, and Vegf production occurs [138].

Research indicates that serum interleukin-6 (*IL-6*) concentration is typically elevated in patients with DMD who are steroid-naïve or untreated compared to those treated with glucocorticoids. [139,140]. Different cell regulators, lncRNAs such as Neat1, H19, Meg3, and lnc-31, maintain control over this gene. The expression of *Il-6* and *Il-8* benefits from increased levels of lncRNA Neat1. This regulation is achieved by downregulating the expression of miR-181c [59], miR-139 [141], and miR-144-3p [142]. Similarly, H19 contributes to the regulation of IL-6 in a comparable manner. It promotes the expression of this gene by acting as a sponge for miR-let-7a, contributing to an increase in vascular inflammation. In contrast, Meg3 and lnc-31 exert inhibitory effects on the secretion of *Tnf*-α and *Il-6* [127,143]. *Vim* represents another gene under the influence of specific lncRNAs. Some studies reveal notably elevated *Vim* gene expression in DMD and dystrophic hearts [144,145], but its exact function is still unclear. Xist lncRNA emerges as a potential regulator, possibly inhibiting miR-92b and freeing miR-92b from the 3′ UTR of *Smad7*. This process potentially triggers the activation and subsequent upregulation of *Vim* expression [146]. Additionally, Zhang et al. found that miR-143 and Malat1 directly regulate the *Vim* and epithelial–cadherin protein levels, but lower miR-143 expression combined with elevated Malat1 [147]. Conversely, higher Neat1 levels lead to an elevation of E-cadherin, Neural–cadherin protein, and Vim protein simultaneously [87]. Instead, the lncRNA H19 acts as a regulator that affects the expression of *Vim*, *Zeb1*, and *Zeb2* by competing with endogenous RNAs, specifically miR-138 and miR-200a [148]. Furthermore, the overexpression of Meg3 leads to downregulation of *Vim* and fibronectin mesenchymal markers as it inhibits gastric cancer cell proliferation [149].

**Figure 3:**
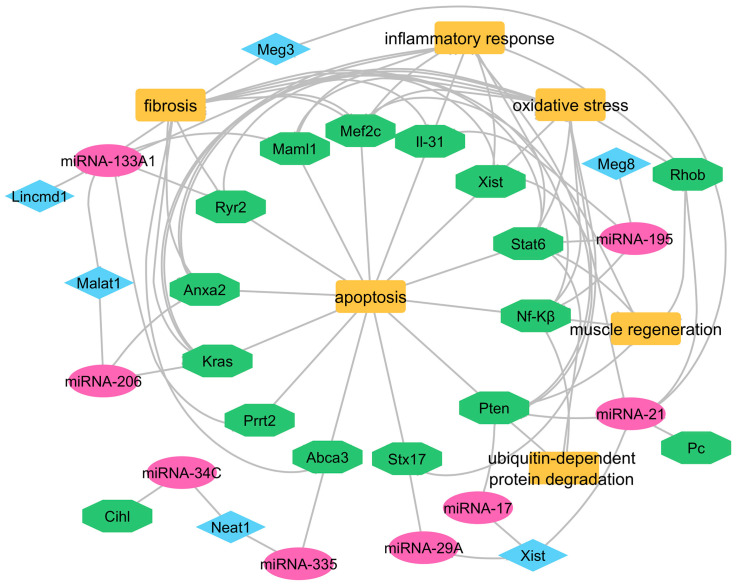
A network diagram illustrating the interactions among miRNAs (pink), genes (green), and lncRNAs (blue), as well as their associations with key biological processes and pathways (orange) (Cytoscape software 3.10.3).

Interactions between lncRNAs and miRNAs are highly significant, as they play a crucial role in regulating gene expression. The expression of muscle-specific miRNAs regulates the modulation of muscle differentiation and homeostasis, while their patterns show changes in conditions like myocardial infarction and DMD, together with other myopathies [150,151] (Table 3, Figure 3). Cell migration and invasion are inhibited through the interaction of miRNA that simultaneously affects the gene expression of *Anxa2* and *Kras*. The removal of miR-206 leads to worse and more rapid symptoms in mouse models of DMD [152]. Mice expressing the mature form of this miRNA demonstrate enhanced muscle tissue repair and a slower progression of Duchenne muscular dystrophy [153]. Also, the overexpression of miR-1 leads to a decrease in Malat1 expression [154]. Interestingly, miR-1 has demonstrated its potential to suppress breast cancer development by downregulating *Kras* and Malat1 transcription [155]. Investigations into miR-1 levels have shown significant elevation in DMD patients compared to healthy control subjects [156,157].

## 6. Conclusions

Recently, the importance of lncRNAs has increased because they can regulate many genes and are master regulators of various genetic, epigenetic, and biological processes essential to developing multiple disorders but are also potent regulators of muscle regeneration, affecting genes involved in DMD disease. The mechanisms of lncRNAs-mediated gene regulation discussed in this manuscript clarify that lncRNAs can regulate DMD-related gene expression at the post-transcriptional level, with a less abundant but significant role in post-translational regulation. Post-transcriptionally, lncRNAs such as Miat, H19, Meg3, and lnc-31 modulate mRNA stability, splicing, translation, and miRNA interactions (e.g., miRNA sponging, direct mRNA binding), influencing myogenic differentiation and DMD progression. Post-translationally, lncRNAs like H19 and FKBP1C regulate protein stability and signaling pathways (e.g., Akt, AMPK), highlighting their critical role in skeletal muscle development and DMD pathology. Adeno-associated virus (AAV)-mediated delivery holds promise for DMD therapy, supported by precedents in other diseases where AAV vectors delivered lncRNAs like H19 (cancer), Malat1 (vascular disease), and Neat1 (neurological disorders) to achieve therapeutic effects.

However, while the presented examples suggest potential roles for lncRNAs in DMD pathogenesis and therapeutic applications, further studies are required to establish causal relationships and validate their therapeutic potential.

## Figures and Tables

**Table 1:** Regulatory effects of lncRNAs on some genes related to muscle biology.

LncRNA	Regulation	Genes	Function of lncRNA	Organism	References
Gm15441	negative	*Txnip*	decline hepatic glucose production	Mouse	[20,22]
3110045C21Rik	positive	*Ddr2*	role in insulin signaling pathway regulation	Mouse	[23]
3110045C21Rik	positive	*Cdh1*	to be involved in fibrosis	Mouse	[24]
3110045C21Rik	negative	*Acta2*	to be involved in fibrosis	Mouse	[24]
3110045C21Rik	negative	*Tgfb1*	to be involved in fibrosis	Mouse	[24]
Lnc_011371	positive	*Mb*, *Clic5*	up-regulation after birth	Anhui white goats (AWG)	[26]
Lnc_007561	positive	*Tcf4*	regulates myogenesis	Anhui white goats (AWG)	[26]
Lnc_001728	positive	*S100 A4*	promotes cardiomyocyte production, inhibiting apoptosis	Anhui white goats (AWG)	[26]
Ppp1r1b	positive	*Prc2*	leads to the induction of myogenic transcription factors	C2C12 and Human skeletal muscle myoblast	[27]
Miat	negative	*Dnmt1*, *Dnmt3a*, *and Dnmt3b*	silenced MIAT leads to reduced cell proliferation and promotes apoptosis	Human breast cancer	[28]
Lnc-1700113A16RIK	positive	*Myog*, *MEF2D*	enhance the differentiation of skeletal muscle stem cells	Mouse	[29]
Lnc-22988, Lnc-372289 and Lnc-482286	positive	*Acta1*, *Eno3*, *Myl1*, *Myom1*, *Myoz1*, *Neb*, *Ryr1*, *and Tnnc2*	could directly or indirectly regulate muscle proliferation, differentiation, and development	Pig	[30]
H19	negative	*Smad1*, *Smad5 and Cdc6*	promotion of skeletal muscle differentiation and regeneration	C2C12 Mouse myoblast cell line	[31]
H19	positive	*Dusp27*	promote AMPK activity in muscle cells and stimulate glucose uptake and mitochondrial biogenesis	Mouse	[32]
Mar1	positive	*Myod*, *Myog*, *Mef2c*, *and Myf5*	positively correlated with muscle differentiation	Mouse	[33]
Syisl	negative	*Ezh2*	regulate muscle atrophy and sarcopenia	C2C12	[34]
LncMyod (Gm45923)	positive	*Imp2*	promoted myoblast proliferation and inhibited myoblast differentiation in the C2C12 cell line	C2C12	[35]
Dum	negative	*Dppa2*	promotes myogenic differentiation	C2C12	[36]
FKBP1C	negative	*Myh1b*	suppress myoblast proliferation and enhance myoblast differentiation in fast and slow muscle fibers.	Chicken	[37]

**Table 3:** Interplay between specific lncRNAs, miRNAs, and genes in the DMD disease.

LncRNA	Effect on Genes	miRNA	Genes	References
LincMD1	negative	miR-133A1	*Mef2c* and *Maml1*	[158]
Meg8	negative	miR-195	*Stat6/Nf-Kβ/Il-31*	[159]
Malat1	negative	miR-133A1	*Ryr2*	[44]
Malat1	negative	miR-206	*Anxa2* and *Kras*	[160]
Xist	negative	miR-17	*Pten*	[132]
Xist	negative	miR-29A	*Stx17*	[122]
Xist	negative	miR-21	*Pc/Xist*	[161]
Neat1	negative	miR-34C	*Cisplatin (DDP)*	[162]
Neat1	negative	miR-335	*Abca3*	[163]
Meg3	positive	miR-133A1	*Prrt2*	[164]
Meg3	negative	miR-21	*Rhob* and *Pten*	[8]

## Data Availability

Information on the data acquired in the project is available upon request from the corresponding author, tomasz_sadkowski@sggw.edu.pl.

## Abbreviations

DMD	Duchenne Muscular Dystrophy
lncRNA	Long Non-Coding RNA
miRNA	MicroRNA
MRFs	Myogenic Regulatory Factors
Myf5	Myogenic Factor 5
MyoD	Myogenic Differentiation Antigen
MyoG	Myogenin
Mrf4	Myogenic Regulatory Factor 4
Mhc	Myosin Heavy Chain
ORF	Open Reading Frame
T2dm	Type 2 Diabetes Mellitus
WGCNA	Weighted Gene Co-expression Network Analysis
Prc2	Polycomb Repressive Complex 2
MuSC	Muscle Stem Cells
TGF-β	Transforming Growth Factor Beta
EMT	Epithelial–Mesenchymal Transition
AMPK	AMP-Activated Protein Kinase
Akt	Protein Kinase B
IGF-1	Insulin-like Growth Factor 1
Pi3k	Phosphoinositide 3-Kinase
P-Akt	Phosphorylated Akt
Vim	Vimentin
IL-6	Interleukin-6
IL-8	Interleukin-8
TNF-α	Tumor Necrosis Factor Alpha
MAPK	Mitogen-Activated Protein Kinase
Dusp27	Dual-Specificity Phosphatase 27
IMP2	IGF2 mRNA-Binding Protein 2
Mrckα	Myotonic dystrophy kinase-related Cdc42-binding kinase alpha
Snca	Alpha-Synuclein
Mbnl1	Muscleblind-Like Splicing Regulator 1
LincRNA	Long Intergenic Non-Coding RNA
Ryr2	Ryanodine Receptor 2
Stx17	Syntaxin 17
N-Ras	Neuroblastoma RAS Viral (v-ras) Oncogene Homolog
c-Myc	MYC Proto-Oncogene
TGFBI	Transforming Growth Factor Beta Induced
Prrt2	Proline-Rich Transmembrane Protein 2
ROCK1	Rho Associated Coiled-Coil Containing Protein Kinase 1
NF-κB	Nuclear Factor kappa-light-chain-enhancer of activated B cells
VEGFA	Vascular Endothelial Growth Factor A
SIRT1	Sirtuin 1
FOXO1	Forkhead Box O1
CXCL12	C-X-C Motif Chemokine Ligand 12
CXCR4	C-X-C Chemokine Receptor Type 4
STAT3	Signal Transducer and Activator of Transcription 3
SOCS6	Suppressor of Cytokine Signaling 6
JAK2	Janus Kinase 2
MEF2C	Myocyte Enhancer Factor 2C
MALAT1	Metastasis Associated Lung Adenocarcinoma Transcript 1
NEAT1	Nuclear Enriched Abundant Transcript 1
MEG3/MEG8	Maternally Expressed Gene 3/8
H19	H19 Imprinted Maternally Expressed Transcript
Lnc-31	Long Non-Coding RNA 31
ST2	Suppression of Tumorigenicity 2 (biomarker)
EZH2	Enhancer of Zeste Homolog 2
BRCA1	Breast Cancer 1

## References

[1] Ousterout D.G., Kabadi A.M., Thakore P.I., Majoros W.H., Reddy T.E., Gersbach C.A. (2015). Multiplex CRISPR/Cas9-Based Genome Editing for Correction of Dystrophin Mutations That Cause Duchenne Muscular Dystrophy. Nat. Commun..

[2] Duan Y., Song B., Zheng C., Zhong Y., Guo Q., Zheng J., Yin Y., Li J., Li F. (2021). Dietary Beta-Hydroxy Beta-Methyl Butyrate Supplementation Alleviates Liver Injury in Lipopolysaccharide-Challenged Piglets. Oxidative Med. Cell. Longev..

[3] Mercuri E., Bönnemann C.G., Muntoni F. (2019). Muscular Dystrophies. Lancet.

[4] Cai A., Kong X. (2019). Development of CRISPR-Mediated Systems in the Study of Duchenne Muscular Dystrophy. Hum. Gene Ther. Methods.

[5] Crisafulli S., Sultana J., Fontana A., Salvo F., Messina S., Trifirò G. (2020). Global Epidemiology of Duchenne Muscular Dystrophy: An Updated Systematic Review and Meta-Analysis. Orphanet J. Rare Dis..

[6] Dowling J.J., Weihl C.C., Spencer M.J. (2021). Molecular and Cellular Basis of Genetically Inherited Skeletal Muscle Disorders. Nat. Rev. Mol. Cell Biol..

[7] Tajbakhsh S. (2009). Skeletal Muscle Stem Cells in Developmental versus Regenerative Myogenesis. J. Intern. Med..

[8] Kumar S., Williams D., Sur S., Wang J.-Y., Jo H. (2019). Role of Flow-Sensitive microRNAs and Long Noncoding RNAs in Vascular Dysfunction and Atherosclerosis. Vasc. Pharmacol..

[9] Rodríguez-Cruz M., Cruz-Guzmán O.D.R., Almeida-Becerril T., Solís-Serna A.D., Atilano-Miguel S., Sánchez-González J.R., Barbosa-Cortés L., Ruíz-Cruz E.D., Huicochea J.C., Cárdenas-Conejo A. (2018). Potential Therapeutic Impact of Omega-3 Long Chain-Polyunsaturated Fatty Acids on Inflammation Markers in Duchenne Muscular Dystrophy: A Double-Blind, Controlled Randomized Trial. Clin. Nutr..

[10] Cheng J.-T., Wang L., Wang H., Tang F.-R., Cai W.-Q., Sethi G., Xin H.-W., Ma Z. (2019). Insights into Biological Role of LncRNAs in Epithelial-Mesenchymal Transition. Cells.

[11] Martone J., Mariani D., Desideri F., Ballarino M. (2019). Non-Coding RNAs Shaping Muscle. Front. Cell Dev. Biol..

[12] Esteller M. (2011). Non-Coding RNAs in Human Disease. Nat. Rev. Genet..

[13] Mattick J.S., Makunin I.V. (2006). Non-Coding RNA. Hum. Mol. Genet..

[14] Derrien T., Johnson R., Bussotti G., Tanzer A., Djebali S., Tilgner H., Guernec G., Martin D., Merkel A., Knowles D.G. (2012). The GENCODE v7 Catalog of Human Long Noncoding RNAs: Analysis of Their Gene Structure, Evolution, and Expression. Genome Res..

[15] Liu W., Kuang H., Xia Y., Pope Z.C., Wang Z., Tang C., Yin D. (2019). Regular Aerobic Exercise-Ameliorated Troponin I Carbonylation to Mitigate Aged Rat Soleus Muscle Functional Recession. Exp. Physiol..

[16] Chouvarine P., Photiadis J., Cesnjevar R., Scheewe J., Bauer U.M.M., Pickardt T., Kramer H.-H., Dittrich S., Berger F., Hansmann G. (2021). RNA Expression Profiles and Regulatory Networks in Human Right Ventricular Hypertrophy Due to High Pressure Load. iScience.

[17] Kallen A.N., Zhou X.-B., Xu J., Qiao C., Ma J., Yan L., Lu L., Liu C., Yi J.-S., Zhang H. (2013). The Imprinted H19 lncRNA Antagonizes Let-7 microRNAs. Mol. Cell.

[18] Mousavi K., Zare H., Dell’Orso S., Grontved L., Gutierrez-Cruz G., Derfoul A., Hager G.L., Sartorelli V. (2013). eRNAs Promote Transcription by Establishing Chromatin Accessibility at Defined Genomic Loci. Mol. Cell.

[19] Volders P.-J., Anckaert J., Verheggen K., Nuytens J., Martens L., Mestdagh P., Vandesompele J. (2019). LNCipedia 5: Towards a Reference Set of Human Long Non-Coding RNAs. Nucleic Acids Res..

[20] Wang L., Fan J., Han L., Qi H., Wang Y., Wang H., Chen S., Du L., Li S., Zhang Y. (2020). The Micropeptide LEMP Plays an Evolutionarily Conserved Role in Myogenesis. Cell Death Dis..

[21] Brocker C.N., Kim D., Melia T., Karri K., Velenosi T.J., Takahashi S., Aibara D., Bonzo J.A., Levi M., Waxman D.J. (2020). Long Non-Coding RNA Gm15441 Attenuates Hepatic Inflammasome Activation in Response to PPARA Agonism and Fasting. Nat. Commun..

[22] Xin M., Guo Q., Lu Q., Lu J., Wang P.-S., Dong Y., Li T., Chen Y., Gerhard G.S., Yang X.-F. (2021). Identification of Gm15441, a Txnip Antisense lncRNA, as a Critical Regulator in Liver Metabolic Homeostasis. Cell Biosci..

[23] Zhang N., Zhou Y., Yuan Q., Gao Y., Wang Y., Wang X., Cui X., Xu P., Ji C., Guo X. (2018). Dynamic Transcriptome Profile in Db/Db Skeletal Muscle Reveal Critical Roles for Long Noncoding RNA Regulator. Int. J. Biochem. Cell Biol..

[24] Arvaniti E., Moulos P., Vakrakou A., Chatziantoniou C., Chadjichristos C., Kavvadas P., Charonis A., Politis P.K. (2016). Whole-Transcriptome Analysis of UUO Mouse Model of Renal Fibrosis Reveals New Molecular Players in Kidney Diseases. Sci. Rep..

[25] Zhang Y., Wang T., Wang Z., Shi X., Jin J. (2025). Functions and Therapeutic Potentials of Long Noncoding RNA in Skeletal Muscle Atrophy and Dystrophy. J. Cachexia Sarcopenia Muscle.

[26] Ling Y., Zheng Q., Sui M., Zhu L., Xu L., Zhang Y., Liu Y., Fang F., Chu M., Ma Y. (2019). Comprehensive Analysis of LncRNA Reveals the Temporal-Specific Module of Goat Skeletal Muscle Development. Int. J. Mol. Sci..

[27] Kang X., Zhao Y., Van Arsdell G., Nelson S.F., Touma M. (2020). Ppp1r1b-lncRNA Inhibits PRC2 at Myogenic Regulatory Genes to Promote Cardiac and Skeletal Muscle Development in Mouse and Human. Rna.

[28] Ge X., Sun T., Zhang Y., Li Y., Gao P., Zhang D., Zhang B., Wang P., Ma W., Lu S. (2022). The Role and Possible Mechanism of the Long Noncoding RNA LINC01260 in Nonalcoholic Fatty Liver Disease. Nutr. Metab..

[29] Kitamura T., Kitamura Y.I., Funahashi Y., Shawber C.J., Castrillon D.H., Kollipara R., DePinho R.A., Kitajewski J., Accili D. (2007). A Foxo/Notch Pathway Controls Myogenic Differentiation and Fiber Type Specification. J. Clin. Investig..

[30] Kousteni S. (2012). FoxO1, the Transcriptional Chief of Staff of Energy Metabolism. Bone.

[31] Vandewalle C., Van Roy F., Berx G. (2009). The Role of the ZEB Family of Transcription Factors in Development and Disease. Cell. Mol. Life Sci. CMLS.

[32] Jou M.-Y., Philipps A.F., Kelleher S.L., Lönnerdal B. (2010). Effects of Zinc Exposure on Zinc Transporter Expression in Human Intestinal Cells of Varying Maturity. J. Pediatr. Gastroenterol. Nutr..

[33] Li D., Hu X., Yu S., Deng S., Yan M., Sun F., Song J., Tang L. (2020). Silence of lncRNA MIAT-Mediated Inhibition of DLG3 Promoter Methylation Suppresses Breast Cancer Progression via the Hippo Signaling Pathway. Cell. Signal..

[34] Chen G., Chen H., Ren S., Xia M., Zhu J., Liu Y., Zhang L., Tang L., Sun L., Liu H. (2019). Aberrant DNA Methylation of mTOR Pathway Genes Promotes Inflammatory Activation of Immune Cells in Diabetic Kidney Disease. Kidney Int..

[35] Yang M., Wang L.I. (2021). MALAT1 Knockdown Protects from Bronchial/Tracheal Smooth Muscle Cell Injury via Regulation of microRNA-133a/Ryanodine Receptor 2 Axis. J. Biosci..

[36] Fu X., Li S., Jia M., Xu B., Yang L., Ma R., Cheng H., Yang W., Hu P. (2022). Myogenesis Controlled by a Long Non-Coding RNA 1700113A16RIK and Post-Transcriptional Regulation. Cell Regen..

[37] Gao P.F., Guo X.H., Du M., Cao G.Q., Yang Q.C., Pu Z.D., Wang Z.Y., Zhang Q., Li M., Jin Y.S. (2017). LncRNA Profiling of Skeletal Muscles in Large White Pigs and Mashen Pigs during Development. J. Anim. Sci..

[38] Dey B.K., Pfeifer K., Dutta A. (2014). The H19 Long Noncoding RNA Gives Rise to microRNAs miR-675-3p and miR-675-5p to Promote Skeletal Muscle Differentiation and Regeneration. Genes Dev..

[39] Geng T., Liu Y., Xu Y., Jiang Y., Zhang N., Wang Z., Carmichael G.G., Taylor H.S., Li D., Huang Y. (2018). H19 lncRNA Promotes Skeletal Muscle Insulin Sensitivity in Part by Targeting AMPK. Diabetes.

[40] García-Padilla C., Domínguez J.N., Aránega A.E., Franco D. (2019). Differential Chamber-Specific Expression and Regulation of Long Non-Coding RNAs during Cardiac Development. Biochim. Biophys. Acta Gene Regul. Mech..

[41] Sartori R., Schirwis E., Blaauw B., Bortolanza S., Zhao J., Enzo E., Stantzou A., Mouisel E., Toniolo L., Ferry A. (2013). BMP Signaling Controls Muscle Mass. Nat. Genet..

[42] Winbanks C.E., Chen J.L., Qian H., Liu Y., Bernardo B.C., Beyer C., Watt K.I., Thomson R.E., Connor T., Turner B.J. (2013). The Bone Morphogenetic Protein Axis Is a Positive Regulator of Skeletal Muscle Mass. J. Cell Biol..

[43] Neppl R.L., Wu C.-L., Walsh K. (2017). lncRNA Chronos Is an Aging-Induced Inhibitor of Muscle Hypertrophy. J. Cell Biol..

[44] Macpherson P.C.D., Farshi P., Goldman D. (2015). Dach2-Hdac9 Signaling Regulates Reinnervation of Muscle Endplates. Development.

[45] Zhang Z.-K., Li J., Guan D., Liang C., Zhuo Z., Liu J., Lu A., Zhang G., Zhang B.-T. (2018). A Newly Identified lncRNA MAR1 Acts as a miR-487b Sponge to Promote Skeletal Muscle Differentiation and Regeneration. J. Cachexia Sarcopenia Muscle.

[46] Jin J., Du M., Wang J., Guo Y., Zhang J., Zuo H., Hou Y., Wang S., Lv W., Bai W. (2022). Conservative Analysis of Synaptopodin-2 Intron Sense-Overlapping lncRNA Reveals Its Novel Function in Promoting Muscle Atrophy. J. Cachexia Sarcopenia Muscle.

[47] Zhang P., Du J., Guo X., Wu S., He J., Li X., Shen L., Chen L., Li B., Zhang J. (2021). LncMyoD Promotes Skeletal Myogenesis and Regulates Skeletal Muscle Fiber-Type Composition by Sponging miR-370-3p. Genes.

[48] Wang L., Zhao Y., Bao X., Zhu X., Kwok Y.K.-Y., Sun K., Chen X., Huang Y., Jauch R., Esteban M.A. (2015). LncRNA Dum Interacts with Dnmts to Regulate Dppa2 Expression during Myogenic Differentiation and Muscle Regeneration. Cell Res..

[49] Yu J.-A., Wang Z., Yang X., Ma M., Li Z., Nie Q. (2021). LncRNA-FKBP1C Regulates Muscle Fiber Type Switching by Affecting the Stability of MYH1B. Cell Death Discov..

[50] Wang S., Jin J., Xu Z., Zuo B. (2019). Functions and Regulatory Mechanisms of lncRNAs in Skeletal Myogenesis, Muscle Disease and Meat Production. Cells.

[51] He L., Chen Y., Hao S., Qian J. (2018). Uncovering Novel Landscape of Cardiovascular Diseases and Therapeutic Targets for Cardioprotection via Long Noncoding RNA-miRNA-mRNA Axes. Epigenomics.

[52] Jin J.J., Lv W., Xia P., Xu Z.Y., Zheng A.D., Wang X.J., Wang S.S., Zeng R., Luo H.M., Li G.L. (2018). Long Noncoding RNA SYISL Regulates Myogenesis by Interacting with Polycomb Repressive Complex 2. Proc. Natl. Acad. Sci. USA.

[53] Gong C., Li Z., Ramanujan K., Clay I., Zhang Y., Lemire-Brachat S., Glass D.J. (2015). A Long Non-Coding RNA, LncMyoD, Regulates Skeletal Muscle Differentiation by Blocking IMP2-Mediated mRNA Translation. Dev. Cell.

[54] Li J., Yang T., Tang H., Sha Z., Chen R., Chen L., Yu Y., Rowe G.C., Das S., Xiao J. (2021). Inhibition of lncRNA MAAT Controls Multiple Types of Muscle Atrophy by Cis- and Trans-Regulatory Actions. Mol. Ther. J. Am. Soc. Gene Ther..

[55] Zheng L., Huang L., Chen Z., Cui C., Zhang R., Qin L. (2021). Magnesium Supplementation Alleviates Corticosteroid-Associated Muscle Atrophy in Rats. Eur. J. Nutr..

[56] Mercer T.R., Dinger M.E., Mattick J.S. (2009). Long Non-Coding RNAs: Insights into Functions. Nat. Rev. Genet..

[57] Loo T.H., Ye X., Chai R.J., Ito M., Bonne G., Ferguson-Smith A.C., Stewart C.L. (2019). The Mammalian LINC Complex Component SUN1 Regulates Muscle Regeneration by Modulating Drosha Activity. eLife.

[58] Liu Y., Wang J., Zhou X., Cao H., Zhang X., Huang K., Li X., Yang G., Shi X. (2020). miR-324-5p Inhibits C2C12 Cell Differentiation and Promotes Intramuscular Lipid Deposition through lncDUM and PM20D1. Mol. Ther. Nucleic Acids.

[59] Huang Q., Huang C., Luo Y., He F., Zhang R. (2018). Circulating lncRNA NEAT1 Correlates with Increased Risk, Elevated Severity and Unfavorable Prognosis in Sepsis Patients. Am. J. Emerg. Med..

[60] Bovolenta M., Erriquez D., Valli E., Brioschi S., Scotton C., Neri M., Falzarano M.S., Gherardi S., Fabris M., Rimessi P. (2012). The DMD Locus Harbours Multiple Long Non-Coding RNAs Which Orchestrate and Control Transcription of Muscle Dystrophin mRNA Isoforms. PLoS ONE.

[61] Xu X., Hao Y., Xiong S., He Z. (2020). Comprehensive Analysis of Long Non-Coding RNA-Associated Competing Endogenous RNA Network in Duchenne Muscular Dystrophy. Interdiscip. Sci. Comput. Life Sci..

[62] Culligan K.G., Mackey A.J., Finn D.M., Maguire P.B., Ohlendieck K. (1998). Role of Dystrophin Isoforms and Associated Proteins in Muscular Dystrophy (Review). Int. J. Mol. Med..

[63] Gargaun E., Falcone S., Solé G., Durigneux J., Urtizberea A., Cuisset J.M., Benkhelifa-Ziyyat S., Julien L., Boland A., Sandron F. (2021). The lncRNA 44s2 Study Applicability to the Design of 45-55 Exon Skipping Therapeutic Strategy for DMD. Biomedicines.

[64] Shen Y., Kim I.-M., Hamrick M., Tang Y. (2023). Uncovering the Gene Regulatory Network of Endothelial Cells in Mouse Duchenne Muscular Dystrophy: Insights from Single-Nuclei RNA Sequencing Analysis. Biology.

[65] Liu Y., Liu C., Zhang A., Yin S., Wang T., Wang Y., Wang M., Liu Y., Ying Q., Sun J. (2019). Down-Regulation of Long Non-Coding RNA MEG3 Suppresses Osteogenic Differentiation of Periodontal Ligament Stem Cells (PDLSCs) through miR-27a-3p/IGF1 Axis in Periodontitis. Aging.

[66] Gehrig S.M., Ryall J.G., Schertzer J.D., Lynch G.S. (2008). Insulin-like Growth Factor-I Analogue Protects Muscles of Dystrophic Mdx Mice from Contraction-Mediated Damage. Exp. Physiol..

[67] Jing X., Han J., Zhang J., Chen Y., Yuan J., Wang J., Neo S., Li S., Yu X., Wu J. (2021). Long Non-Coding RNA MEG3 Promotes Cisplatin-Induced Nephrotoxicity through Regulating AKT/TSC/mTOR-Mediated Autophagy. Int. J. Biol. Sci..

[68] Peter A.K., Crosbie R.H. (2006). Hypertrophic Response of Duchenne and Limb-Girdle Muscular Dystrophies Is Associated with Activation of Akt Pathway. Exp. Cell Res..

[69] Li Y., Zhang S., Zhang C., Wang M. (2020). LncRNA MEG3 Inhibits the Inflammatory Response of Ankylosing Spondylitis by Targeting miR-146a. Mol. Cell. Biochem..

[70] Messina S., Vita G.L., Aguennouz M., Sframeli M., Romeo S., Rodolico C., Vita G. (2011). Activation of NF-kB Pathway in Duchenne Muscular Dystrophy: Relation to Age. Acta Myol..

[71] Gu L., Zhang J., Shi M., Zhan Q., Shen B., Peng C. (2017). lncRNA MEG3 Had Anti-Cancer Effects to Suppress Pancreatic Cancer Activity. Biomed. Pharmacother..

[72] Anderson J., Seol H., Hathout Y., Spurney C. (2015). Elevated St2 Serum Levels A Biomarker for Cardiomyopathy in Duchenne Muscular Dystrophy. J. Am. Coll. Cardiol..

[73] Zhang D., Qin H., Leng Y., Li X., Zhang L., Bai D., Meng Y., Wang J. (2018). LncRNA MEG3 Overexpression Inhibits the Development of Diabetic Retinopathy by Regulating TGF-Β1 and VEGF. Exp. Ther. Med..

[74] Ishitobi M., Haginoya K., Zhao Y., Ohnuma A., Minato J., Yanagisawa T., Tanabu M., Kikuchi M., Iinuma K. (2000). Elevated Plasma Levels of Transforming Growth Factor Β1 in Patients with Muscular Dystrophy. NeuroReport.

[75] Zhou Y., Zhang X., Klibanski A. (2012). MEG3 Noncoding RNA: A Tumor Suppressor. J. Mol. Endocrinol..

[76] Saito T., Yamamoto Y., Matsumura T., Fujimura H., Shinno S. (2009). Serum Levels of Vascular Endothelial Growth Factor Elevated in Patients with Muscular Dystrophy. Brain Dev..

[77] Zhang Y., Zou Y., Wang W., Zuo Q., Jiang Z., Sun M., De W., Sun L. (2015). Down-Regulated Long Non-Coding RNA MEG3 and Its Effect on Promoting Apoptosis and Suppressing Migration of Trophoblast Cells. J. Cell. Biochem..

[78] Wang M., Huang T., Luo G., Huang C., Xiao X., Wang L., Jiang G., Zeng F. (2015). Long Non-Coding RNA MEG3 Induces Renal Cell Carcinoma Cells Apoptosis by Activating the Mitochondrial Pathway. J. Huazhong Univ. Sci. Technol. Med. Sci..

[79] Li Y., Jiang J., Liu W., Wang H., Zhao L., Liu S., Li P., Zhang S., Sun C., Wu Y. (2018). microRNA-378 Promotes Autophagy and Inhibits Apoptosis in Skeletal Muscle. Proc. Natl. Acad. Sci. USA.

[80] Zhu X., Wu Y.-B., Zhou J., Kang D.-M. (2016). Upregulation of lncRNA MEG3 Promotes Hepatic Insulin Resistance via Increasing FoxO1 Expression. Biochem. Biophys. Res. Commun..

[81] Liu W., Liu X., Luo M., Liu X., Luo Q., Tao H., Wu D., Lu S., Jin J., Zhao Y. (2017). dNK Derived IFN-γ Mediates VSMC Migration and Apoptosis via the Induction of LncRNA MEG3: A Role in Uterovascular Transformation. Placenta.

[82] von Moers A., Zwirner A., Reinhold A., Brückmann O., van Landeghem F., Stoltenburg-Didinger G., Schuppan D., Herbst H., Schuelke M. (2005). Increased mRNA Expression of Tissue Inhibitors of Metalloproteinase-1 and -2 in Duchenne Muscular Dystrophy. Acta Neuropathol..

[83] Yang N.-Q., Luo X.-J., Zhang J., Wang G.-M., Guo J.-M. (2016). Crosstalk between Meg3 and miR-1297 Regulates Growth of Testicular Germ Cell Tumor through PTEN/PI3K/AKT Pathway. Am. J. Transl. Res..

[84] Yue F., Song C., Huang D., Narayanan N., Qiu J., Jia Z., Yuan Z., Oprescu S.N., Roseguini B.T., Deng M. (2021). PTEN Inhibition Ameliorates Muscle Degeneration and Improves Muscle Function in a Mouse Model of Duchenne Muscular Dystrophy. Mol. Ther..

[85] Huang S., Xu Y., Ge X., Xu B., Peng W., Jiang X., Shen L., Xia L. (2019). Long Noncoding RNA NEAT1 Accelerates the Proliferation and Fibrosis in Diabetic Nephropathy through Activating Akt/mTOR Signaling Pathway. J. Cell. Physiol..

[86] Wang L., Yang D., Tian R., Zhang H. (2019). NEAT1 Promotes Retinoblastoma Progression via Modulating miR-124. J. Cell. Biochem..

[87] Zhang Y., Yao X.-H., Wu Y., Cao G.-K., Han D. (2020). LncRNA NEAT1 Regulates Pulmonary Fibrosis through miR-9-5p and TGF-β Signaling Pathway. Eur. Rev. Med. Pharmacol. Sci..

[88] Chen Y.-W., Zhao P., Borup R., Hoffman E.P. (2000). Expression Profiling in the Muscular Dystrophies. J. Cell Biol..

[89] Ding X.-M., Zhao L.-J., Qiao H.-Y., Wu S.-L., Wang X.-H. (2019). Long Non-Coding RNA-P21 Regulates MPP+-Induced Neuronal Injury by Targeting miR-625 and Derepressing TRPM2 in SH-SY5Y Cells. Chem. Biol. Interact..

[90] Ma M., Hui J., Zhang Q., Zhu Y., He Y., Liu X. (2018). Long Non-Coding RNA Nuclear-Enriched Abundant Transcript 1 Inhibition Blunts Myocardial Ischemia Reperfusion Injury via Autophagic Flux Arrest and Apoptosis in Streptozotocin-Induced Diabetic Rats. Atherosclerosis.

[91] Bai Y., Lv Y., Wang W., Sun G., Zhang H. (2018). LncRNA NEAT1 Promotes Inflammatory Response and Induces Corneal Neovascularization. J. Mol. Endocrinol..

[92] Zhang P., Cao L., Zhou R., Yang X., Wu M. (2019). The lncRNA Neat1 Promotes Activation of Inflammasomes in Macrophages. Nat. Commun..

[93] Tu J., Zhao Z., Xu M., Lu X., Chang L., Ji J. (2018). NEAT1 Upregulates TGF-Β1 to Induce Hepatocellular Carcinoma Progression by Sponging Hsa-Mir-139-5p. J. Cell. Physiol..

[94] Chang S., Chen B., Wang X., Wu K., Sun Y. (2017). Long Non-Coding RNA XIST Regulates PTEN Expression by Sponging miR-181a and Promotes Hepatocellular Carcinoma Progression. BMC Cancer.

[95] Yang J., Shen Y., Yang X., Long Y., Chen S., Lin X., Dong R., Yuan J. (2019). Silencing of Long Noncoding RNA XIST Protects against Renal Interstitial Fibrosis in Diabetic Nephropathy via microRNA-93-5p-Mediated Inhibition of CDKN1A. Am. J. Physiol.-Ren. Physiol..

[96] Sun J., Zhang Y. (2019). LncRNA XIST Enhanced TGF-Β2 Expression by Targeting miR-141-3p to Promote Pancreatic Cancer Cells Invasion. Biosci. Rep..

[97] McLennan I.S., Koishi K. (1997). Cellular Localisation of Transforming Growth Factor-Beta 2 and -Beta 3 (TGF-Beta2, TGF-Beta3) in Damaged and Regenerating Skeletal Muscles. Dev. Dyn..

[98] Zhang Y., Wang J., Zhang Y., Wei J., Wu R., Cai H. (2019). Overexpression of Long Noncoding RNA Malat1 Ameliorates Traumatic Brain Injury Induced Brain Edema by Inhibiting AQP4 and the NF-κB/IL-6 Pathway. J. Cell. Biochem..

[99] Jimi T., Wakayama Y., Matsuzaki Y., Hara H., Inoue M., Shibuya S. (2004). Reduced Expression of Aquaporin 4 in Human Muscles with Amyotrophic Lateral Sclerosis and Other Neurogenic Atrophies. Pathol. Res. Pract..

[100] Han Y., Wu Z., Wu T., Huang Y., Cheng Z., Li X., Sun T., Xie X., Zhou Y., Du Z. (2016). Tumor-Suppressive Function of Long Noncoding RNA MALAT1 in Glioma Cells by Downregulation of MMP2 and Inactivation of ERK/MAPK Signaling. Cell Death Dis..

[101] Pan F., Zhu L., Lv H., Pei C. (2016). Quercetin Promotes the Apoptosis of Fibroblast-like Synoviocytes in Rheumatoid Arthritis by Upregulating lncRNA MALAT1. Int. J. Mol. Med..

[102] Wu X.-S., Wang X.-A., Wu W.-G., Hu Y.-P., Li M.-L., Ding Q., Weng H., Shu Y.-J., Liu T.-Y., Jiang L. (2014). MALAT1 Promotes the Proliferation and Metastasis of Gallbladder Cancer Cells by Activating the ERK/MAPK Pathway. Cancer Biol. Ther..

[103] Sun X., Luo L., Li J. (2020). LncRNA MALAT1 Facilitates BM-MSCs Differentiation into Endothelial Cells via Targeting miR-206/VEGFA Axis. Cell Cycle.

[104] Loufrani L., Dubroca C., You D., Li Z., Levy B., Paulin D., Henrion D. (2004). Absence of Dystrophin in Mice Reduces NO-Dependent Vascular Function and Vascular Density: Total Recovery after a Treatment with the Aminoglycoside Gentamicin. Arterioscler. Thromb. Vasc. Biol..

[105] Ji D.-G., Guan L.-Y., Luo X., Ma F., Yang B., Liu H.-Y. (2018). Inhibition of MALAT1 Sensitizes Liver Cancer Cells to 5-Flurouracil by Regulating Apoptosis through IKKα/NF-κB Pathway. Biochem. Biophys. Res. Commun..

[106] Aguennouz M., Vita G.L., Messina S., Cama A., Lanzano N., Ciranni A., Rodolico C., Di Giorgio R.M., Vita G. (2011). Telomere Shortening Is Associated to TRF1 and PARP1 Overexpression in Duchenne Muscular Dystrophy. Neurobiol. Aging.

[107] Zheng S., Zhang X., Wang X., Li J. (2019). MIR31HG Promotes Cell Proliferation and Invasion by Activating the Wnt/β-Catenin Signaling Pathway in Non-Small Cell Lung Cancer. Oncol. Lett..

[108] Feron M., Guevel L., Rouger K., Dubreil L., Arnaud M.-C., Ledevin M., Megeney L.A., Cherel Y., Sakanyan V. (2009). PTEN Contributes to Profound PI3K/Akt Signaling Pathway Deregulation in Dystrophin-Deficient Dog Muscle. Am. J. Pathol..

[109] Cao L., Jiang H., Yang J., Mao J., Wei G., Meng X., Zang H. (2021). LncRNA MIR31HG Is Induced by Tocilizumab and Ameliorates Rheumatoid Arthritis Fibroblast-like Synoviocyte-Mediated Inflammation via miR-214-PTEN-AKT Signaling Pathway. Aging.

[110] Wu Y., Jiang Y., Liu Q., Liu C.-Z. (2019). lncRNA H19 Promotes Matrix Mineralization through Up-Regulating IGF1 by Sponging miR-185-5p in Osteoblasts. BMC Mol. Cell Biol..

[111] Chen X., Yang J., Shen H., Zhang X., Wang H., Wu G., Qi Y., Wang L., Xu W. (2021). Muc5ac Production Inhibited by Decreased lncRNA H19 via PI3K/Akt/NF-kB in Asthma. J. Asthma Allergy.

[112] Wang Z.-M., Xia S.-W., Zhang T., Wang Z.-Y., Yang X., Kai J., Cheng X.-D., Shao J.-J., Tan S.-Z., Chen A.-P. (2020). LncRNA-H19 Induces Hepatic Stellate Cell Activation via Upregulating Alcohol Dehydrogenase III-Mediated Retinoic Acid Signals. Int. Immunopharmacol..

[113] Hou J., Wang L., Wu Q., Zheng G., Long H., Wu H., Zhou C., Guo T., Zhong T., Wang L. (2018). Long Noncoding RNA H19 Upregulates Vascular Endothelial Growth Factor A to Enhance Mesenchymal Stem Cells Survival and Angiogenic Capacity by Inhibiting miR-199a-5p. Stem Cell Res. Ther..

[114] Hu Z., Liu X., Guo J., Zhuo L., Chen Y., Yuan H. (2021). Knockdown of lncRNA MEG8 Inhibits Cell Proliferation and Invasion, but Promotes Cell Apoptosis in Hemangioma, via miR-203-induced Mediation of the Notch Signaling Pathway. Mol. Med. Rep..

[115] Vieira N.M., Elvers I., Alexander M.S., Moreira Y.B., Eran A., Gomes J.P., Marshall J.L., Karlsson E.K., Verjovski-Almeida S., Lindblad-Toh K. (2015). Jagged 1 Rescues the Duchenne Muscular Dystrophy Phenotype. Cell.

[116] Sui S., Sun L., Zhang W., Li J., Han J., Zheng J., Xin H. (2021). LncRNA MEG8 Attenuates Cerebral Ischemia After Ischemic Stroke Through Targeting miR-130a-5p/VEGFA Signaling. Cell. Mol. Neurobiol..

[117] Cabianca D.S., Casa V., Bodega B., Xynos A., Ginelli E., Tanaka Y., Gabellini D. (2012). A Long ncRNA Links Copy Number Variation to a Polycomb/Trithorax Epigenetic Switch in FSHD Muscular Dystrophy. Cell.

[118] Castiglioni I., Caccia R., Garcia-Manteiga J.M., Ferri G., Caretti G., Molineris I., Nishioka K., Gabellini D. (2018). The Trithorax Protein Ash1L Promotes Myoblast Fusion by Activating Cdon Expression. Nat. Commun..

[119] Dimartino D., Colantoni A., Ballarino M., Martone J., Mariani D., Danner J., Bruckmann A., Meister G., Morlando M., Bozzoni I. (2018). The Long Non-Coding RNA Lnc-31 Interacts with Rock1 mRNA and Mediates Its YB-1-Dependent Translation. Cell Rep..

[120] Li Y., Zhang Y., Hu Q., Egranov S.D., Xing Z., Zhang Z., Liang K., Ye Y., Pan Y., Chatterjee S.S. (2021). Functional Significance of Gain-of-Function H19 lncRNA in Skeletal Muscle Differentiation and Anti-Obesity Effects. Genome Med..

[121] Tran T.H.T., Zhang Z., Yagi M., Lee T., Awano H., Nishida A., Okinaga T., Takeshima Y., Matsuo M. (2013). Molecular Characterization of an X(P21.2;Q28) Chromosomal Inversion in a Duchenne Muscular Dystrophy Patient with Mental Retardation Reveals a Novel Long Non-Coding Gene on Xq28. J. Hum. Genet..

[122] Zhou M., Liu X., Qiukai E., Shang Y., Zhang X., Liu S., Zhang X. (2021). Long Non-Coding RNA Xist Regulates Oocyte Loss via Suppressing miR-23b-3p/miR-29a-3p Maturation and Upregulating STX17 in Perinatal Mouse Ovaries. Cell Death Dis..

[123] You L., Wang N., Yin D., Wang L., Jin F., Zhu Y., Yuan Q., De W. (2016). Downregulation of Long Noncoding RNA Meg3 Affects Insulin Synthesis and Secretion in Mouse Pancreatic Beta Cells. J. Cell. Physiol..

[124] Butchart L.C., Terrill J.R., Rossetti G., White R., Filipovska A., Grounds M.D. (2018). Expression Patterns of Regulatory RNAs, Including lncRNAs and tRNAs, during Postnatal Growth of Normal and Dystrophic (Mdx) Mouse Muscles, and Their Response to Taurine Treatment. Int. J. Biochem. Cell Biol..

[125] Bier A., Berenstein P., Kronfeld N., Morgoulis D., Ziv-Av A., Goldstein H., Kazimirsky G., Cazacu S., Meir R., Popovtzer R. (2018). Placenta-Derived Mesenchymal Stromal Cells and Their Exosomes Exert Therapeutic Effects in Duchenne Muscular Dystrophy. Biomaterials.

[126] Dong J., Xia R., Zhang Z., Xu C. (2021). lncRNA MEG3 Aggravated Neuropathic Pain and Astrocyte Overaction through Mediating miR-130a-5p/CXCL12/CXCR4 Axis. Aging.

[127] Luo R., Jin H., Li L., Hu Y.-X., Xiao F. (2020). Long Noncoding RNA MEG3 Inhibits Apoptosis of Retinal Pigment Epithelium Cells Induced by High Glucose via the miR-93/Nrf2 Axis. Am. J. Pathol..

[128] Xiao F., Li L., Fu J.-S., Hu Y.-X., Luo R. (2020). Regulation of the miR-19b-Mediated SOCS6-JAK2/STAT3 Pathway by lncRNA MEG3 Is Involved in High Glucose-Induced Apoptosis in hRMECs. Biosci. Rep..

[129] Zhong J., Jiang L., Huang Z., Zhang H., Cheng C., Liu H., He J., Wu J., Darwazeh R., Wu Y. (2017). The Long Non-Coding RNA Neat1 Is an Important Mediator of the Therapeutic Effect of Bexarotene on Traumatic Brain Injury in Mice. Brain Behav. Immun..

[130] Yong H., Wu G., Chen J., Liu X., Bai Y., Tang N., Liu L., Wei J. (2020). lncRNA MALAT1 Accelerates Skeletal Muscle Cell Apoptosis and Inflammatory Response in Sepsis by Decreasing BRCA1 Expression by Recruiting EZH2. Mol. Ther. Nucleic Acids.

[131] Zhang Y., Yuan Q., Wei Q., Li P., Zhuang Z., Li J., Liu Y., Zhang L., Hong Z., He W. (2022). Long Noncoding RNA XIST Modulates microRNA-135/CREB1 Axis to Influence Osteogenic Differentiation of Osteoblast-like Cells in Mice with Tibial Fracture Healing. Hum. Cell.

[132] Zhang X., Wu H., Mai C., Qi Y. (2020). Long Noncoding RNA XIST/miR-17/PTEN Axis Modulates the Proliferation and Apoptosis of Vascular Smooth Muscle Cells to Affect Stanford Type A Aortic Dissection. J. Cardiovasc. Pharmacol..

[133] Contreras O., Rebolledo D.L., Oyarzún J.E., Olguín H.C., Brandan E. (2016). Connective Tissue Cells Expressing Fibro/Adipogenic Progenitor Markers Increase under Chronic Damage: Relevance in Fibroblast-Myofibroblast Differentiation and Skeletal Muscle Fibrosis. Cell Tissue Res..

[134] Zhang Q., Chen B., Liu P., Yang J. (2018). XIST Promotes Gastric Cancer (GC) Progression through TGF-Β1 via Targeting miR-185. J. Cell. Biochem..

[135] Mo Y., He L., Lai Z., Wan Z., Chen Q., Pan S., Li L., Li D., Huang J., Xue F. (2018). LINC01287/miR-298/STAT3 Feedback Loop Regulates Growth and the Epithelial-to-Mesenchymal Transition Phenotype in Hepatocellular Carcinoma Cells. J. Exp. Clin. Cancer Res. CR.

[136] Dill T.L., Carroll A., Pinheiro A., Gao J., Naya F.J. (2021). The Long Noncoding RNA Meg3 Regulates Myoblast Plasticity and Muscle Regeneration through Epithelial-Mesenchymal Transition. Development.

[137] Lv D., Bi Q., Li Y., Deng J., Wu N., Hao S., Zhao M. (2021). Long Non-coding RNA MEG3 Inhibits Cell Migration and Invasion of Non-small Cell Lung Cancer Cells by Regulating the miR-21-5p/PTEN Axis. Mol. Med. Rep..

[138] Shen S., Ma L., Shao F., Jin L., Bian Z. (2020). Long Non-Coding RNA (lncRNA) NEAT1 Aggravates Cerebral Ischemia-Reperfusion Injury by Suppressing the Inhibitory Effect of miR-214 on PTEN. Med. Sci. Monit. Int. Med. J. Exp. Clin. Res..

[139] Mammen A.L., Sartorelli V. (2015). IL-6 Blockade as a Therapeutic Approach for Duchenne Muscular Dystrophy. EBioMedicine.

[140] Wang Q., Liu S., Wang H., Liu L., Zhang S., Ming Y., Zhao Y., Cheng K. (2021). Silencing Long Noncoding RNA NEAT1 Alleviates Acute Liver Failure via the EZH2-Mediated microRNA-139/PUMA Axis. Aging.

[141] Wei J.-L., Wu C.-J., Chen J.-J., Shang F.-T., Guo S.-G., Zhang X.-C., Liu H. (2020). LncRNA NEAT1 Promotes the Progression of Sepsis-Induced Myocardial Cell Injury by Sponging miR-144-3p. Eur. Rev. Med. Pharmacol. Sci..

[142] Sun Y., Zhong L., He X., Wang S., Lai Y., Wu W., Song H., Chen Y., Yang Y., Liao W. (2019). LncRNA H19 Promotes Vascular Inflammation and Abdominal Aortic Aneurysm Formation by Functioning as a Competing Endogenous RNA. J. Mol. Cell. Cardiol..

[143] Sun J., Song X., Su L., Cao S. (2018). Long Non-Coding RNA LncHIFCAR Promotes Osteoarthritis Development via Positively Regulating HIF-1α and Activating the PI3K/AKT/mTOR Pathway. Int. J. Clin. Exp. Pathol..

[144] Rayavarapu S., Coley W., Cakir E., Jahnke V., Takeda S., Aoki Y., Grodish-Dressman H., Jaiswal J.K., Hoffman E.P., Brown K.J. (2013). Identification of Disease Specific Pathways Using in Vivo SILAC Proteomics in Dystrophin Deficient Mdx Mouse. Mol. Cell. Proteomics MCP.

[145] Guo Z., Geng M., Huang Y., Han G., Jing R., Lin C., Zhang X., Zhang M., Fan G., Wang F. (2022). Upregulation of Wilms’ Tumor 1 in Epicardial Cells Increases Cardiac Fibrosis in Dystrophic Mice. Cell Death Differ..

[146] Tam C., Wong J.H., Tsui S.K.W., Zuo T., Chan T.F., Ng T.B. (2019). LncRNAs with miRNAs in Regulation of Gastric, Liver, and Colorectal Cancers: Updates in Recent Years. Appl. Microbiol. Biotechnol..

[147] Zhang L., Niyazi H.E.X.D., Zhao H.R., Cao X.P., Abudula M.N.S., Ye W.J., Zhang S.A., Yiming R.H.M., Zhang Y., Su W.P. (2017). Effects of miRNA-143 and the Non-Coding RNA MALAT1 on the Pathogenesis and Metastasis of HeLa Cells. Genet. Mol. Res. GMR.

[148] Li C., Zhou L., He J., Fang X.-Q., Zhu S.-W., Xiong M.-M. (2016). Increased Long Noncoding RNA SNHG20 Predicts Poor Prognosis in Colorectal Cancer. BMC Cancer.

[149] Jiao J., Zhang S. (2019). Long Non-coding RNA MEG-3 Suppresses Gastric Carcinoma Cell Growth, Invasion and Migration via EMT Regulation. Mol. Med. Rep..

[150] Alexander M.S., Casar J.C., Motohashi N., Vieira N.M., Eisenberg I., Marshall J.L., Gasperini M.J., Lek A., Myers J.A., Estrella E.A. (2014). MicroRNA-486–Dependent Modulation of DOCK3/PTEN/AKT Signaling Pathways Improves Muscular Dystrophy–Associated Symptoms. J. Clin. Investig..

[151] Cacchiarelli D., Martone J., Girardi E., Cesana M., Incitti T., Morlando M., Nicoletti C., Santini T., Sthandier O., Barberi L. (2010). MicroRNAs Involved in Molecular Circuitries Relevant for the Duchenne Muscular Dystrophy Pathogenesis Are Controlled by the Dystrophin/nNOS Pathway. Cell Metab..

[152] Tang Y., Xiao G., Chen Y., Deng Y. (2018). LncRNA MALAT1 Promotes Migration and Invasion of Non-Small-Cell Lung Cancer by Targeting miR-206 and Activating Akt/mTOR Signaling. Anticancer Drugs.

[153] Liu N., Williams A.H., Maxeiner J.M., Bezprozvannaya S., Shelton J.M., Richardson J.A., Bassel-Duby R., Olson E.N. (2012). microRNA-206 Promotes Skeletal Muscle Regeneration and Delays Progression of Duchenne Muscular Dystrophy in Mice. J. Clin. Investig..

[154] Jin C., Yan B., Lu Q., Lin Y., Ma L. (2016). Reciprocal Regulation of Hsa-miR-1 and Long Noncoding RNA MALAT1 Promotes Triple-Negative Breast Cancer Development. Tumour Biol..

[155] Saliani M., Mirzaiebadizi A., Javadmanesh A., Siavoshi A., Ahmadian M.R. (2022). KRAS-Related Long Noncoding RNAs in Human Cancers. Cancer Gene Ther..

[156] Li X., Li Y., Zhao L., Zhang D., Yao X., Zhang H., Wang Y.-C., Wang X.-Y., Xia H., Yan J. (2014). Circulating Muscle-Specific miRNAs in Duchenne Muscular Dystrophy Patients. Mol. Ther. Nucleic Acids.

[157] Hu J., Kong M., Ye Y., Hong S., Cheng L., Jiang L. (2014). Serum miR-206 and Other Muscle-Specific microRNAs as Non-Invasive Biomarkers for Duchenne Muscular Dystrophy. J. Neurochem..

[158] Jin C.F., Li Y., Ding X.B., Li X., Zhang L.L., Liu X.F., Guo H. (2017). Lnc133b, a Novel, Long Non-Coding RNA, Regulates Bovine Skeletal Muscle Satellite Cell Proliferation and Differentiation by Mediating miR-133b. Gene.

[159] Roohaninasab M., Yavari S.F., Babazadeh M., Hagh R.A., Pazoki M., Amrovani M. (2022). Evaluating the Role of lncRNAs in the Incidence of Cardiovascular Diseases in Androgenetic Alopecia Patients. Cardiovasc. Toxicol..

[160] Wang S.-H., Zhang W.-J., Wu X.-C., Zhang M.-D., Weng M.-Z., Zhou D., Wang J.-D., Quan Z.-W. (2016). Long Non-Coding RNA Malat1 Promotes Gallbladder Cancer Development by Acting as a Molecular Sponge to Regulate miR-206. Oncotarget.

[161] Dong Y., Wan G., Peng G., Yan P., Qian C., Li F. (2020). Long Non-Coding RNA XIST Regulates Hyperglycemia-Associated Apoptosis and Migration in Human Retinal Pigment Epithelial Cells. Biomed. Pharmacother..

[162] Hu Y., Yang Q., Wang L., Wang S., Sun F., Xu D., Jiang J. (2018). Knockdown of the Oncogene lncRNA NEAT1 Restores the Availability of miR-34c and Improves the Sensitivity to Cisplatin in Osteosarcoma. Biosci. Rep..

[163] Zhang M., Guo J., Liu L., Huang M., Li Y., Bennett S., Xu J., Zou J. (2022). The Role of Long Non-Coding RNA, Nuclear Enriched Abundant Transcript 1 (NEAT1) in Cancer and Other Pathologies. Biochem. Genet..

[164] Yao C., Guo G., Huang R., Tang C., Zhu Q., Cheng Y., Kong L., Ren J., Fang M. (2022). Manual Therapy Regulates Oxidative Stress in Aging Rat Lumbar Intervertebral Discs through the SIRT1/FOXO1 Pathway. Aging.

